# First person – Anna Fassler Bakhman

**DOI:** 10.1242/dmm.052933

**Published:** 2026-04-27

## Abstract

First Person is a series of interviews with the first authors of a selection of papers published in Disease Models & Mechanisms, helping researchers promote themselves alongside their papers. Anna Fassler Bakhman is first author on ‘ Systematic structure-based analysis of RET variants in MEN2A and Hirschsprung's disease, and the paradoxical co-occurrence of both conditions’, published in DMM. Anna conducted the research described in this article while a PhD student in Prof. Mickey Kosloff's lab at University of Haifa. She is now a postdoc in the lab of Prof. Mickey Kosloff at University of Haifa, investigating computational structural biology of protein signalling and mechanisms of interactions.

**Figure:**
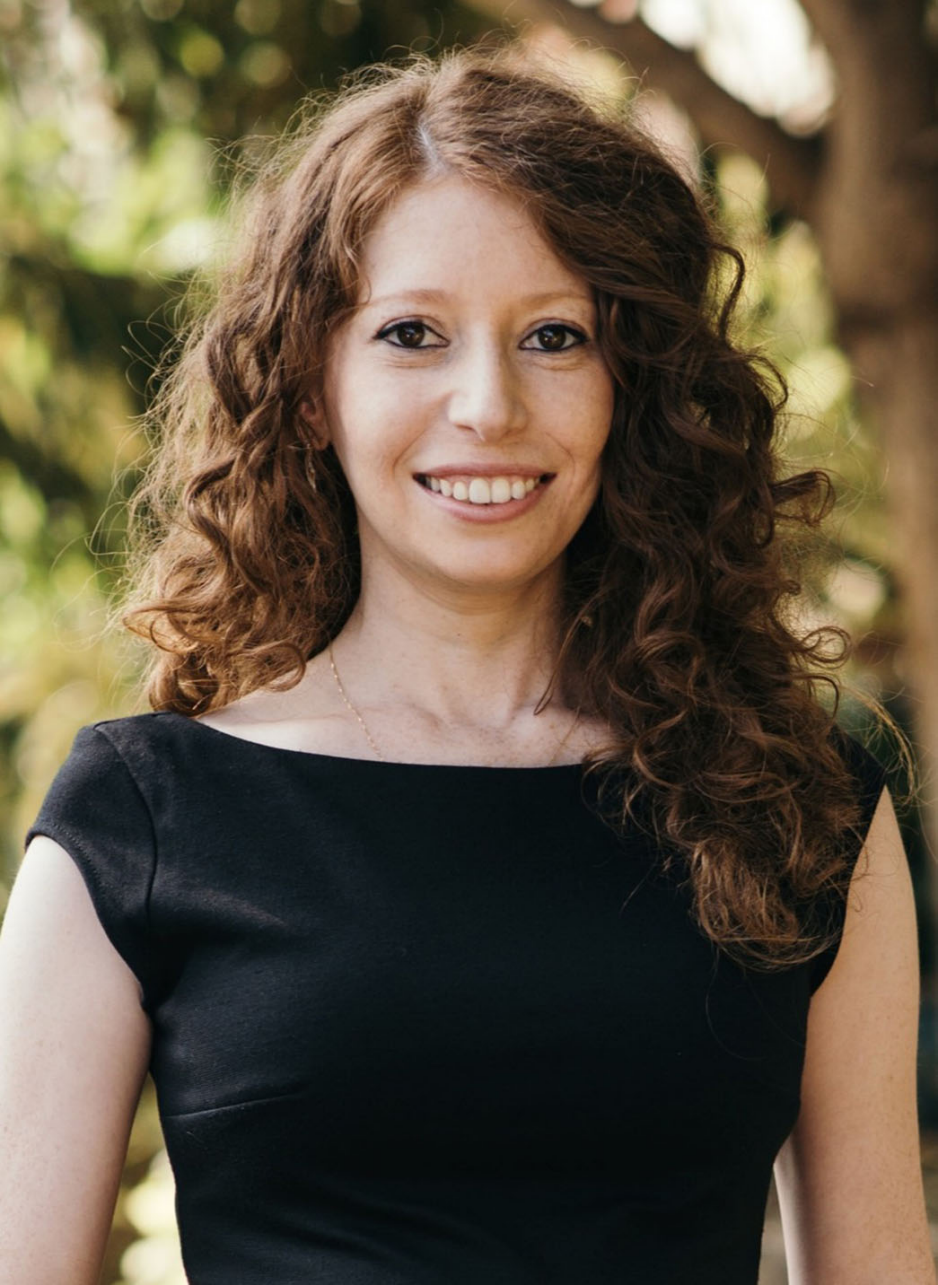
Anna Fassler Bakhman

**Who or what inspired you to become a scientist?**

My curiosity about biology began early. From my first biology lessons in elementary school, I was fascinated by how complex living systems work and how small molecular changes can have profound effects on health and disease. As a teenager, I volunteered for four years as a first-aid ambulance responder, where I encountered a wide range of medical situations and saw how biological processes directly affect people's lives.

During my B.Sc. I joined an excellence program that included actual research in a laboratory. This experience sparked my fascination with how protein structures determine biological function, and discovering how computational tools allow us to explore such mechanisms at the molecular level inspired me to pursue research in structural and computational biology.

By integrating 3D structural analysis with computational modelling, we identified key molecular determinants that influence signalling and protein–protein interactions, explaining how specific mutations can lead to distinct disease outcomes.

**What is the main question or challenge in disease biology you are addressing in this paper? How did you go about investigating your question or challenge?**

Many diseases arise from mutations or variants in proteins that alter how these proteins interact with other molecules in the cell. In our study, we addressed a particularly intriguing challenge, i.e. what are the structural mechanisms underlying the paradoxical effects of mutations associated with MEN2A and Hirschsprung's disease (HSCR), which can affect the same signalling pathway differently.

To investigate this question, we used computational structural biology approaches, analysing protein structures and interactions. By integrating 3D structural analysis with computational modelling, we identified key molecular determinants that influence signalling and protein–protein interactions, explaining how specific mutations can lead to distinct disease outcomes.

**How would you explain the main findings of your paper to non-scientific family and friends?**

Our cells rely on proteins that need to ‘talk’ to each other to control key processes, such as tissue growth and development. Sometimes, a single small change within one of these proteins can disrupt such cellular communication and lead to disease. We examined a specific protein called RET, which controls how some cells grow and develop. Variations in this RET protein can lead to two very different diseases. One is Hirschsprung's disease, where abnormal development of nerves in the intestine can cause severe digestive problems. The other disease is MEN2A, a hereditary syndrome that increases the risk of developing cancer, especially in the thyroid gland. By closely examining the 3D shape of the RET protein and how it can interact with signalling partners, we were able to understand how these small variations in RET can disrupt its communication in opposite ways. Our paper explains why these ‘opposite’ diseases occur, and provides clues that can eventually help scientists develop better ways to diagnose and treat these diseases.

**What are the potential implications of these results for disease biology and the possible impact on patients?**

Understanding the structural mechanisms behind RET variants explains how some genetic changes disrupt early development, leading to Hirschsprung's disease, while other variants cause excessive signalling that leads to cancers, such as MEN2A. By revealing the molecular principles that underlie and distinguish these outcomes, our work enables to interpret RET variants identified in genetic testing and can help clinicians better assess disease risk and guide clinical outcomes. Importantly, these findings also suggest mutation-specific therapeutic strategies. For example, molecules that stabilize RET can, potentially, mitigate the loss-of-function effects in Hirschsprung's disease, while therapies that reduce receptor activation may help with MEN2A. Ultimately, linking specific mutations to their structural and functional consequences can support precise diagnosis, risk stratification and the development of targeted treatments for patients with RET-associated diseases.

Linking specific mutations to their structural and functional consequences can support precise diagnosis, risk stratification and the development of targeted treatments for patients with RET-associated diseases.

**Figure:**
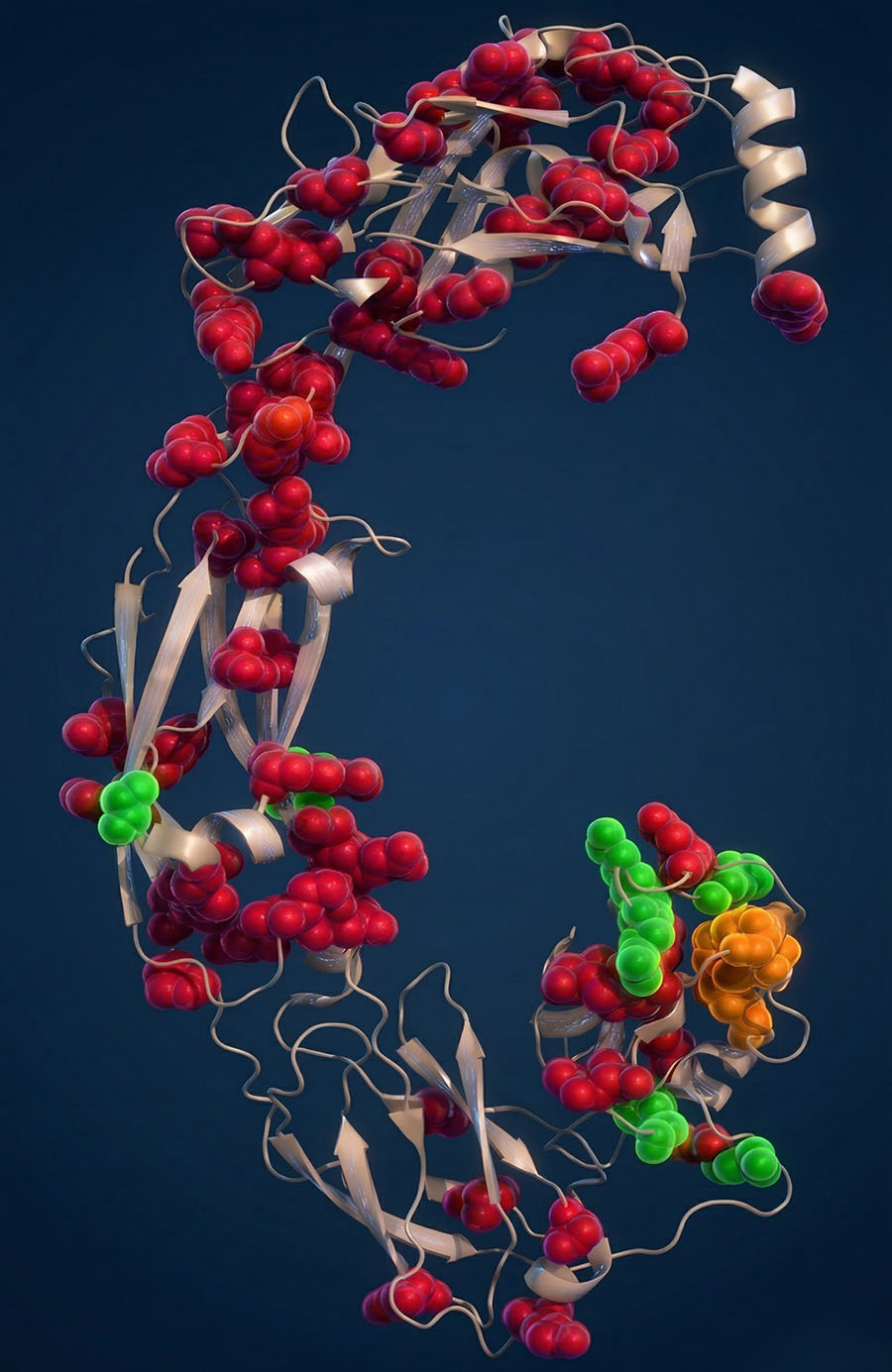
Mapping of 77 mutations in the RET receptor associated with Hirschsprung's disease, MEN2A or both onto the 3D structure of RET.

**Why did you choose DMM for your paper?**

DMM focuses on connecting fundamental biological mechanisms with disease processes, which aligns closely with the goals of our work. The journal provides an excellent platform for studies that combine mechanistic insights with relevance to disease biology.

**Given your current role, what challenges do you face and what changes could improve the professional lives of other scientists in this role?**

One of the main difficulties at this stage is the instability of the academic job market. As postdocs, we are expected to focus on producing strong science while, simultaneously, trying to secure our next career step, which can be stressful and distracting. Supportive mentors, colleagues and collaborative environments make a big difference in navigating this period. Greater interaction between academia and industry could also help researchers build professional networks and become more aware of the diverse career paths available outside the traditional academic track.

**What's next for you?**

I plan to continue studying the structural mechanisms that control protein signalling and interactions using computational approaches, with a stronger focus on clinical implications. Ultimately, I am interested in understanding how molecular mechanisms translate into diseases and how enhancing our knowledge of these mechanisms can contribute to improved diagnostics and therapeutic strategies.

**Tell us something interesting about yourself that wouldn't be on your CV**

Outside the lab, I enjoy baking and am constantly searching for new and challenging recipes to try. Experimenting in the kitchen is a creative outlet for me and a way to unwind. I find it surprisingly similar to research: it involves curiosity, experimentation, and a bit of trial and error before everything comes together perfectly.
